# The Weekly Ward Review by the Consultant on the General Adult Inpatient Wards in Mersey Care NHS Foundation Trust – How Are We Doing? Do We Need to Improve the Patient Experience?

**DOI:** 10.1192/bjo.2025.10374

**Published:** 2025-06-20

**Authors:** Ankita Vinjamuri, Jonathan Tjong A Tjoe, Ranjan Baruah, Roweida Sammour, Declan Hyland

**Affiliations:** Mersey Care NHS Foundation Trust, Liverpool, United Kingdom

## Abstract

**Aims:** Ward reviews are a pivotal component of patient care in psychiatric inpatient settings, offering a structured opportunity for patients to engage with their treatment team and contribute to their treatment plan. Ward reviews are essential for discussing treatment progress, making necessary adjustments to care plans, and addressing patient concerns. The aim of this quality improvement project was to assess and improve the experience of ward reviews for inpatients on the Trust’s general adult inpatient wards by ensuring inpatients are well-informed about the timing of and attendees for their ward reviews and help them prepare effectively for their ward review.

**Methods:** Inpatients on one of the Trust’s general adult inpatient wards were asked to complete a survey to determine their level of awareness of when their weekly ward review with the Consultant or Higher Trainee was due to happen and of what would be discussed in the ward review. The survey also captured whether the patient was aware of who would be present for their ward review and how prepared they felt. Finally, they were asked how comfortable they would feel with writing down their thoughts and feelings about their ward review before it happened and whether they thought this would be useful.

**Results:** 17 of the 20 patients on the general adult inpatient ward completed the survey. Half of the patients indicated that they knew when their next ward review was. Approximately 25% of patients knew which members of staff would be present in their ward review. 40% of the patients said they felt prepared for their ward review. Overall satisfaction with the ward review was low at only 40%. 60% of the patients stated they would feel comfortable with writing down their thoughts and feelings ahead of their ward review about what to discuss in the review.

**Conclusion:** The authors identified that, given that patient awareness of when their ward review was due to take place and patient satisfaction with the ward review was low, this needed to be addressed and improved. A “ward review sign” stating the day and time of the next ward review was designed and put up in each patient’s bedroom. A ward review template was designed to capture what things the patient wished to discuss in their ward review and the nursing team were asked to support patients with completing this in preparation for their next ward review. The impact of these interventions should then be evaluated.

